# Primary care provider perceptions of intake transition records and shared care with outpatient cardiac rehabilitation programs

**DOI:** 10.1186/1472-6963-11-231

**Published:** 2011-09-23

**Authors:** Jonathan Yee, Karen Unsworth, Neville Suskin, Robert D Reid, Veronica Jamnik, Sherry L Grace

**Affiliations:** 1 School of Kinesiology and Health Science, York University, 4700 Keele Street, Toronto, Ontario, Canada; 2 London Health Sciences Centre, London, Ontario, Canada; 3 Minto Prevention and Rehabilitation Centre, University of Ottawa Heart Institute, Ottawa, Ontario, Canada; 4 Department of Women's Health, Toronto General Hospital, Toronto, Ontario, Canada

## Abstract

**Background:**

While it is recommended that records are kept between primary care providers (PCPs) and specialists during patient transitions from hospital to community care, this communication is not currently standardized. We aimed to assess the transmission of cardiac rehabilitation (CR) program intake transition records to PCPs and to explore PCPs' needs in communication with CR programs and for intake transition record content.

**Method:**

144 PCPs of consenting enrollees from 8 regional and urban Ontario CR programs participated in this cross-sectional study. Intake transition records were tracked from the CR program to the PCP's office. Sixty-six PCPs participated in structured telephone interviews.

**Results:**

Sixty-eight (47.6%) PCPs received a CR intake transition record. Fifty-eight (87.9%) PCPs desired intake transition records, with most wanting it transmitted via fax (n = 52, 78.8%). On a 5-point Likert scale, PCPs strongly agreed that the CR transition record met their needs for providing patient care (4.32 ± 0.61), with 48 (76.2%) reporting that it improved their management of patients' cardiac risk. PCPs rated the following elements as most important to include in an intake transition record: clinical status (4.67 ± 0.64), exercise test results (4.61 ± 0.52), and the proposed patient care plan (4.59 ± 0.71).

**Conclusions:**

Less than half of intake transition records are reaching PCPs, revealing a large gap in continuity of patient care. PCP responses should be used to develop an evidence-based intake transition record, and procedures should be implemented to ensure high-quality transitional care.

## Background

Cardiovascular disease (CVD) is a leading cause of mortality and morbidity in the developed world [1]. Indeed, while many patients are surviving and living with CVD, substantial health risks continue following cardiac events and procedures. The standard of in-patient cardiac care in clinical practice guidelines in many countries [2-5] consists of referral to cardiac rehabilitation (CR) and facilitation of post-discharge primary care provider (PCP) follow-up. Certainly, shared care [6] is particularly important to support risk reduction in the period following cardiac hospitalization, to minimize this risk of recurrent or adverse events. For instance, Ahmed et al. [7] showed that following cardiac hospitalization, shared care between generalists and cardiac specialists resulted in significantly improved left ventricular function evaluation, increased prescription rates for ACE-inhibitors, and lower odds of 90-day readmission (OR = 0.54) for heart failure patients when compared to patients treated by either type of physician alone.

Cardiac patients may be referred from primary care to cardiac specialists, admitted to hospital for a cardiac event or procedure, and referred to and participate in CR, and for each of these transitions it is recommended that a record is sent to primary care. Indeed, an intake and discharge transition record should be generated and sent from the CR program to the providers involved in the patient's care [8]. This is intended to ensure that a patient's continuing health needs are identified and communicated to PCPs for ongoing shared management. These transition records are central to inter-organizational or provider communication [9], which can result in improved delivery and quality of care [10], decreased fragmentation [11], and perhaps fewer hospital readmissions [12]. Inadequate inter-provider communication forces health care providers at the next phase of patient care to spend time and resources compiling missing material and duplicating previously gathered patient-related information [11]. For instance, a study by Smith et al. reported that, in 25.6% of primary care visits, the PCP spent 5-10 minutes unsuccessfully searching for missing clinical information, with 10.4% of visits resulting in 10 or more minutes of searching [13]. Providers must be contacted to validate information, or must rely on patient recall. This is costly, can result in inappropriate utilization of health care resources, and even patient management errors [14].

Intake CR transition records in particular notify PCPs that patients have entered a CR program, communicate preliminary clinical information on which the CR plan is based, and engage physicians in the shared care of the patient early in the CR process. Our previous research has shown that only 42% of PCPs receive a CR discharge transition record [15], but no studies to our knowledge have investigated the flow and content of CR intake transition records to primary care. Overall, best practice in inpatient-to-outpatient care transition communication is not currently standardized (although a consensus policy has been developed), and little is known about outpatient-to-outpatient transition communication [16]. As such, it is unclear if PCPs actually perceive early communication with CR programs to be needed to support quality care, and if they do, what information PCPs need and how they would like to receive it. Thus, the objectives of this study were to: (1) assess the transmission of CR intake transition records to PCPs, and (2) explore PCP needs for communication with CR programs and intake transition record content.

## Methods

### Design and Procedure

This study was observational and cross-sectional in design. The following CR sites and institutions participated in this study and ethics approval was obtained (with Research ethics board [REB] number given if applicable): Alexandra Hospital, Grey Bruce Health Services, London Health Sciences Centre (REB# 15218E), Southlake Regional Health Centre (REB# 252-2010), University Health Network (REB# 08-0439-AE), University of Ottawa Heart Institute (REB# 2008401-01H), Ross Memorial Hospital, York Central Hospital (no REB#), and York University (REB #2008-096). CR is an outpatient chronic disease management program designed to improve and maintain cardiovascular health through individualized, inter-professional care. CR programs offer medical assessment, structured exercise, client and family education, as well as comprehensive risk factor and behavior modification. Consecutive enrollees from each participating CR site were approached to solicit consent at their intake appointment by a staff member involved in the patient's care. In Ontario, on average patients undergo supervised exercise 1-2 times per week, over a median of 5 months (manuscript under review, *JCRP*). Each of the 8 CR sites were chosen to represent a diversity of CR programs from large academic to smaller regional sites (see Table 1). Recruitment occurred between September, 2008 and July, 2010. Consenting participants were asked to complete a brief survey which assessed sociodemographic and clinical characteristics. They were also asked to provide the name of their PCP, which could be a general practitioner or nurse-practitioner.

**Table 1:** Descriptive Characteristics of Cardiac Rehabilitation Programs and Surrounding Region

CR Program Location	Transition Record Transmission Mode	Annual CR Patient Volume	# PCPs in Region	# Full-time CR Staff	Length of CR Program (months)	Hospital Type
London	Electronic	400	335	8	6	Academic

Ottawa	Paper	2100	1150	30	2+	Academic

Ingersoll	Paper	100	55	~2	6-8	Community

Owen Sound	Paper	160	65	~2	5	Community

Thornhill	Paper	410	600	5	3-6	Community

Newmarket	Paper	300	260	8	6	Community

Lindsay	Paper	80	25	~3	3	Community

CR = cardiac rehabilitation; PCP = primary care provider.

The PCP's contact information and characteristics were extracted from the College of Physicians and Surgeons of Ontario directory (CPSO; http://www.cpso.on.ca/docsearch/), which is a publically-accessible online physician database. This information was used to mail the PCP an information letter and consent form, including an option to participate in a structured telephone interview to provide their perceptions of CR intake communication.

Intake transition records were created by each CR program individually at various time points prior to study inception as part of their program development. One site's record was changed from paper to electronic transmission after the program committed to participate in the study, but the content was not altered. The records were mailed (n = 6 sites) or sent electronically (n = 1 site) to the PCPs according to usual practice at the CR program. One CR program did not generate intake transition records as they only sent discharge records to PCPs.

Where the PCP provided consent, a modified version of Dillman's tailored design approach [17] was used to verify receipt of the intake transition records. Administrative assistants were asked to check 'yes' or 'no' whether the intake summary was in the patients' file. This was achieved by multiple contacts to the PCP's office, as follows: 1) a verification form sent by mail, 2) replacement fax to non-responders 1 week later, and finally 3) telephone contact to non-responders 2 weeks later. Once the intake transition record was confirmed as "received" or "not received", a telephone interview to explore PCP perceptions of transition record content was scheduled with PCPs who indicated willingness on their consent form.

### Participants

The only inclusion criterion for PCPs was that their patient was deemed eligible to complete the CR program following intake assessment. The sole exclusion criterion was that the PCP had not already consented to participate in the study based on consent of another one of their patients referred to CR, in order to avoid duplicate PCP interviews.

### Measures

To describe the patient sample, the patient survey included items assessing sociodemographic and clinical characteristics through forced-choice response options. The PCP's sex, year of graduation, and location of medical school was extracted from CPSO to describe the physician sample. In addition, PCPs were asked to rate their satisfaction with previous CR transition records received on a 5-point Likert scale (1 = very unsatisfied to 5 = extremely satisfied).

Receipt of a CR intake transition record was recorded as 'yes' or 'no' by the PCP's administrative staff, where 'no' was also recorded in the instance of non-response. For electronic intake summaries, receipt confirmation occurred passively as access to the eSummary was captured within the CR program database.

Structured interviews were conducted to address the second research question regarding PCPs' perceptions of communication with CR. The interview guide was developed based on available literature, input by the investigative team and participating CR programs, and questions from our previous study [15]. The interview guide was finalized based on responses to pilot interviews with PCPs [18]. Sixty-four consenting PCPs who also agreed to a brief interview were contacted within a month of receiving the intake confirmation receipt. Structured interviews were conducted by telephone to ensure convenience for PCPs. The interview guide was comprised of forced-choice questions that explored the PCPs overall perception of and needs in communication with CR programs, transmission mode preferences, use of intake transition records in patient care, and preferred intake transition record content. Some questions included an additional open-ended probe for further description. If the PCP had received their patient's intake transition record from the CR program, they were additionally asked about their views of the specific intake transition record received.

### Analyses

All analyses were performed using SPSS version 17.0 [19]. Interviews were audiotaped, transcribed verbatim (except to preserve anonymity), and entered into SPSS for quantitative analysis. A second coder verified that all transcripts were transcribed and coded accurately.

A descriptive examination of CR patient and PCP characteristics was performed. PCP characteristics were compared based on participation status using one-way analysis of variance (ANOVA) and χ^2 ^as appropriate, with post-hoc tests of Least Significant Difference (LSD) where the former was significant.

To assess the first objective, the rate of intake transition records received was calculated by dividing the number received (numerator) by the number of PCPs (denominator) attached to consenting CR patients. This rate was re-calculated counting only transition records confirmed to be sent by CR as the denominator. The file was stratified by CR site, and the range of transition record receipt by site was described and compared using chi-square.

For the second objective, a descriptive analysis of interview responses was performed. Independent samples t-tests and Pearson correlations were used to determine whether PCP perceptions of intake transition records differed by their sociodemographic characteristics and also by patient characteristics. Finally, ANOVA was used to test for significant differences in perceptions by CR site.

## Results

### Respondent Characteristics

**Figure 1:**
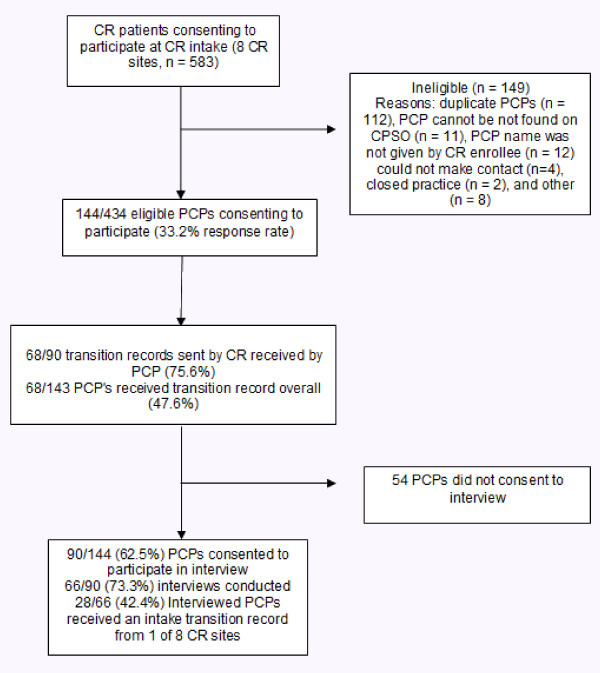
**Diagram of study flow**.

A study flow diagram illustrating the accrual of CR patient participants and their corresponding PCPs is shown in Figure 1. Of the 583 CR enrollees who consented to participate, 402 (76.1%) were male, and the mean age was 61.5 (SD = 11.4) years. The patient sample had a self-reported previous history of the following cardiovascular conditions or procedures: myocardial infarction (n = 222; 42.6%), percutaneous coronary intervention (n = 196; 37.6%), stable angina (n = 151; 29.0%), coronary artery bypass surgery (n = 122; 23.4%), arrhythmia (n = 29; 5.6%), stroke (n = 27, 5.2%), and heart failure, (n = 21, 4.0%). Sixty-five (12.7%) CR enrollees did not report a history of the above cardiac conditions or procedures, and were likely referred for other non-cardiac chronic conditions (e.g., diabetes, renal disease, pulmonary disease) considering trends in the province toward integrated chronic disease management programs.

One hundred and forty-four of the 434 (33.2%) eligible PCPs consented to this study. The consenting PCPs included 3 (2.1%) nurse practitioners, while all others were family physicians. Table 2 displays and compares PCP characteristics by participant status. Participating PCPs were significantly more likely to be female and to have graduated from medical school more recently than declining PCPs. PCPs were quite satisfied with CR transition records they had received in the past (mean = 3.51 ± 1.20 on a 5-point Likert scale, with higher scores indicating greater satisfaction). The number of PCPs per CR site where their patient received care ranged from 4 to 63.

**Table 2:** Descriptive Characteristics of Primary Care Providers by Participant Status, N = 583

Characteristic	Participants (%)			Non-Participants (%)	
	Interviewed N = 66	Total N = 144	p	Declined N = 330	p*
Sex, Male	35 (53.0)	80 (55.6)	0.62	240 (72.7)	< 0.001
Location of Medical School			0.33		0.14
Ontario	50 (78.1)	102 (72.3)		223 (67.8)	
Canada, non-Ontario	8 (12.5)	20 (14.2)		37 (11.2)	
Outside Canada	6 (9.4)	19 (13.5)		69 (21.0)	
Year of Graduation From Medical School (Mean ± SD)	1984 ± 10.74	1986 ± 10.25	0.03	1982 ± 10.85	0.001
Previous Satisfaction with CR Transition records† (Mean ± SD)	3.68 ± 1.15	3.51 ± 1.20	0.16	-	-

Three PCPs are nurse-practitioners. The majority of ineligible PCPs were ineligible due to duplication, and therefore their characteristics are not shown.

*test for significant differences between total participating PCPs and declining PCPs.

† Scores range from 1-5, with higher scores denoting greater satisfaction.

Overall, 90 of 144 (62.5%) participating PCPs consented to be interviewed. Interviews were successfully conducted with 66 (73.3%) of these PCPs. As displayed in Table 2, there were no significant differences between PCPs who participated in an interview and those who did not, even with regard to degree of satisfaction with previously-received CR transition records. The number of interviewed PCPs per CR site where their patient received care ranged from 2 to 32. Overall, 28 (43.8%) interviewed PCPs received a CR intake transition record. The number of interviewed PCPs per CR site who received an intake summary ranged from 1 to 11.

### PCP Receipt of CR Intake Transition Records

Overall, 143 of 144 (99.3%) intake transition records were verified, with only one unverified record recorded as "no". Sixty-eight (47.6%) CR intake transition records were received by consenting PCP offices. Verification of un-received intake transition records with CR sites revealed that only 90 (62.9%) intake transition records were sent by the CR site to the PCP. Of the 53 intake transition records not sent by CR, one CR site only sends intake transition records when a within-program physician does not see the patient (34/143 records = 23.8%), one site does not send any intake transition records (15/143 records = 10.5%), and four (2.8%) patients' intake records were not sent because the patient did not proceed with CR. Thus, 75.6% (68/90) of intake transition records sent by CR were received by the PCP. Office staff were asked to report whether or not the PCP had read the summary, 67 (46.9%) reported yes, 75 (52.4%) reported no, and 1 (0.1%) were unsure.

Pearson Chi-square was computed to explore variability in CR intake receipt by CR program. There was significant variation in CR intake transition report receipt by CR site (*p *< 0.001). When excluding the site that did not send intake transition records, the number and percent of intake transition records received by PCPs for each site were as follows: University Health Network (4/4, 100%); London Health Sciences Centre (9/11;81.8%); York Central Hospital (18/25;72.0%); Southlake Hospital (5/5; 50.0%); Alexandra Hospital (2/4;50.0%); University of Ottawa Health Institute (22/63;34.9%).

Most PCPs (n = 58, 87.9%) were informed by the patient themselves that they were enrolled in CR following referral, while 16 (24.2%) became aware when they received the referral documentation through the cardiac specialist. Five (7.7%) PCPs stated that receiving the intake transition record was the sole way they learned that their patient had enrolled in CR.

#### Do PCPs Want a CR Intake Transition record and if so, Why?

Fifty-nine (89.4%) PCPs agreed that they wanted to receive an intake transition record when their patient enrolled in CR. When asked why in open-ended fashion, 11 (18.9%) PCPs responded that the intake transition records serve as indication that their patient has enrolled in a CR program, 13 (22.4%) responded that it provides comprehensive documentation of the patient's cardiac status, and 8 (13.8%) PCPs stated that the intake transition record provides additional and new patient information. When asked whether or not they would like to receive a transition record at other points along the continuum of care, 64 (97.0%) PCPs responded that they would also like to receive a transition record at CR discharge, and 43 (65.2%) would like to receive an interim report when clinically indicated. PCPs were not more likely to want a discharge than intake transition record (p > .05).

When asked in which format they would like to receive intake communication, 52 (78.8%) PCPs responded fax was acceptable, 38 (57.6%) mail, and 17 (25.8%) via web or email. For those who did not prefer electronic transition records, when asked why in open-ended fashion, 15 (22.7%) PCPs stated that they had not adopted electronic technologies in their practice, and 9 (14.1%) PCPs reported that they preferred a hard copy which they scan into an incompatible electronic health record (EHR).

Sixty (92.3%) PCPs agreed that receiving an intake transition record fostered a sense of shared care with CR. Forty-eight (76.2%) PCPs reported that receiving a CR intake transition report improved their management of their patients' cardiac risk. Fifty-six (86.2%) PCPs reported that they are useful for patient care. When asked why in open-ended fashion, 13 (19.7%) PCPs responded that it provides baseline information so the PCP can track patient progress, 10 (15.1%) PCPs reported they use it as an indication of the patient's CR enrollment, and 9 (13.6%) reported that it provides a comprehensive summary of all risk areas, some of which the PCP chart may not contain. Nineteen (29.7%) PCPs reported that receiving an intake transition record affects the number of visits they have with a patient.

### What Form & Content do PCPs Prefer for the CR Intake Transition Record?

When asked what is the optimal length for an intake transition record, PCPs desired a mean of 1.33 (SD = 0.66) pages (median = 1.00). When asked who should receive an intake transition record, 66 (100.0%) PCPs responded that they should receive a transition record, 58 (87.9%) responded the specialist should also receive a transition record, and 53 (80.3%) responded that a copy should also be given to the patient.

PCP preferences for specific intake transition record data elements are presented in descending order of importance in Table 3. PCPs rated clinical status, exercise test results (with exercise training recommendations/prescription), and identification of risk factors that were not controlled to guideline-established [8] targets as the most important information to be included, while non-modifiable risk factors and subjective reports were rated as least important. When asked if there was any other information that they would like included in the transition records (open-ended response), 1 (1.5%) PCP responded that they wanted patient attendance records, whereas another PCP (1.5%) asked for additional information about the patient's functional status.

**Table 3:** PCP Ratings of Perceived Importance of Data Elements in CR Intake Transition records, N = 66

CR Transition record Data Element	Importance Rating **(Mean **± **SD)**
Clinical status (e.g., NYHA/CCS class)	4.67 ± 0.64
Exercise test results and recommendations	4.61 ± 0.52
Patient care plan	4.59 ± 0.70
Current medications	4.50 ± 0.85
Risk factor "flags" where patient does not meet guideline target	4.35 ± 0.87
Blood pressure	4.12 ± 0.94
Smoking status	4.11 ± 1.04
Lipids	4.09 ± 1.06
Psychological well-being (i.e., anxiety and depression scores)	3.94 ± 0.99
BMI and waist circumference	3.86 ± 1.04
Nutrition	3.77 ± 0.96
Description of the CR program	3.76 ± 1.04
Subjective reports of patient motivation and participation during CR assessment	3.62 ± 1.00
Non-modifiable risk factors	3.24 ± 1.20

Ratings were made on a 5-point Likert scale, from 1 being 'not important at all to include in the intake transition record' to 5 'being extremely important to include in the intake transition record'.

BMI, body mass index; PCP, primary care provider; NYHA, New York Heart Association; CCS, Canadian Cardiovascular Society; CR, cardiac rehabilitation

When comparing information needs by PCP characteristics, it was found that female PCPs rated smoking status (*p *= 0.005) and off-target indicators (*p *= 0.003) as significantly more important to include in the intake transition record than male PCPs. Moreover, more recent medical school graduation was positively correlated with selecting smoking (r = 0.29, *p *= 0.02), off-target indicators (r = 0.43, *p *< 0.001), and current medications (r = 0.27, *p *= 0.03) as desired content. PCPs who graduated in a medical school outside of Canada rated patient subjective reports during CR as more important to include than PCPs who graduated from a school in Canada. (p = 0.04).

### PCP Perceptions of CR Intake Transition Records Received

Table 4 displays PCP perceptions of intake transition records they received for their patients, in descending order. Overall, PCPs perceived the quality of the intake transition records as high. An ANOVA was computed to explore variability in perceptions by CR program. The mean overall rating (SD) ranged from 3.50 (2.12) to 4.71 (0.49; *p *> 0.05).

**Table 4:** PCP Perceptions of CR Intake Transition records Received, N = 28

Quality Indicator	Rating **(Mean **± **SD)**
All necessary information was included in the intake transition record	4.46 ± 0.79
The intake transition record was of high quality	4.43 ± 0.69
I was satisfied with the organization of the intake transition record	4.32 ± 0.77
The intake transition record met my needs	4.32 ± 0.61
The intake transition record was easy to understand	4.32 ± 0.77
I was satisfied with the length of the intake transition record	4.25 ± 0.97
I was satisfied with the length of time required to read the intake transition record	4.11 ± 0.88
Receiving an intake transition record from CR makes me more likely to refer my patients to the program	4.04 ± 1.10
I was satisfied with the time from patient referral to transition record receipt	3.96 ± 0.84
I used information in the intake transition record to manage my patient's risk factors	3.89 ± 1.03
I will likely go back to the information in the intake transition record again at a later time and use it for patient care	3.82 ± 0.98
I used the information in the intake transition record to:	
Manage my patient's medications	3.61 ± 1.10
Manage my patient's exercise habits	3.39 ± 1.20
Manage my patient's weight	3.11 ± 0.96
The transition record included non-useful information	2.36 ± 0.95

Ratings were made on a 5-point Likert scale, with 1 being the lowest quality or greatest disagreement and 5 being the highest quality or greatest agreement.

PCP, primary care provider; CR, cardiac rehabilitation.

When comparing perceptions of intake transition records received by PCP characteristics, it was found that there were no significant differences in PCP responses by sex, medical school graduation year, or location of medical school from which the PCP graduated (*p *> 0.05). When comparing PCP transition record perceptions by patient characteristics, it was found that when providing care to female patients, PCPs rated the usefulness of the intake transition records for exercise (*p *= 0.02) and weight management (*p *= 0.04) information as higher than when caring for male patients.

## Discussion

Previous evidence regarding care transitions between secondary or tertiary and primary health care has shown that receipt, timeliness, and content of communication is often suboptimal [20-24]. This study investigated early shared care communication processes between CR and PCPs. Similar to Riley et al.'s study which showed only 42% transmission of CR discharge transition records to PCPs [15], results herein revealed that less than half of PCPs received an intake transition record for their patients. There were significant differences in transmission rate by CR program, suggesting lack of standardized processes. However, given that almost all PCPs want to receive an intake record so they have a comprehensive summary of their patient's health which they can track over time, clearly standardization is warranted. Moreover, most PCPs were not aware their patients had enrolled in a CR program until they were notified by the patient. Overall, PCPs prefer to receive a concise (less than 1.5 page-long) faxed intake transition record that includes clinical status, exercise test results, and risk factor status in relation to evidence-based targets.

### CR Intake Transition Record Transmission

PCPs were quite satisfied with the timeliness with which they received the transition records. However, less than half of PCPs received the intake transition records. Moreover, there was wide variation in PCP receipt of CR intake transition records based on CR site, ranging from 0-100%. The lack of PCP receipt of CR intake transition records can be partially explained by one CR site only sending an intake transition record if there was no follow-up from an in-house specialist, and another CR site not sending CR intake transition records at all. As well, only approximately three-quarters of intake transition records that CR programs confirmed were sent to PCPs, were received. This may have been due to the practice at some CR programs to send the transition records only to the specialist who refers the patient, rather than identifying all relevant providers [14].

This reality contrasts sharply with the results of this study and previous work by our group [15] which suggest that almost all PCPs want to receive CR transition records. Over three-quarters of PCPs report they use the information within the record to manage their patients' risk. In particular, PCPs used the information for managing their patient's medications, followed by exercise habits and weight. The PCPs use the information as an indicator of patient enrollment, to track change in clinical and risk factor status, and to ensure they have a comprehensive summary of a patient's health. The latter likely provides impetus for the initial referral, as CR programs offer inter-professional care which cannot be replicated by a PCP alone. Clearly, professional and community standards need to be developed, and processes should be established to promote effective transition communication [8,15].

Over three-quarters of PCPs desired transition records via fax, and only one-quarter desired them via electronic means. None of the CR programs transmitted via fax, and this would likely increase timeliness of PCP receipt. Format incompatibility of information sent by CR and stored in PCP offices was identified as a barrier to optimal transitional care. At this time, many PCPs are in the process of adopting EHRs and/or prefer hard copies of intake transition records. The cardiac healthcare community needs to develop standard transmission protocols to optimize transitional inter-provider communication.

### CR Intake Transition Record Content

Interviews with PCPs enabled feedback regarding their needs for transitional care. PCPs were highly satisfied with both previous CR transition records received, as well as with the quality of the intake records received for patients in the study. There was, however, a noticeable range in the degree of satisfaction of intake reports by CR site. A number of possible reasons could explain this variation, which may include the size and type of CR site, the site location, or even differences in the presentation of the intake reports. To pinpoint the reasons for this variation requires a more robust study design (i.e. a cluster randomized trial). In addition, PCPs were highly satisfied with the organization of the intake reports, and perceived they could easily understand all the content as presented. Overall, the transition records met PCP needs, made PCPs more willing to refer future patients, and were often used by PCPs for reference.

The PCPs expressed a desire for concise (less than 1.5 pages) intake transition records. PCPs were highly satisfied with the length of transition records received, and the time required to read them. Data elements that PCPs consider as most pertinent to include in intake transition records were the clinical status of the patient, exercise stress test results, patient care plan, and the medication list. Similar findings were reported in studies examining discharge transition records [15,25,26], where the clinical diagnosis and list of medications taken were considered more important details when compared to other data elements. Elements that were considered least important to PCPs were non-modifiable risk factors and subjective reports of the patient's level of motivation or emotions. However, the recent Transitions of Care Consensus Policy Statement, recommended that a section of the transition record be devoted to communicating "a patient's preferences, priorities, goals and values" [16]. Whether this information is perceived as unimportant by PCPs because they rely on their own interactions with patients or patient reports as a source of this information deserves future investigation.

#### Other Patient-level, Clinical and Policy Implications

Over 80% of PCPs reported that CR intake transition records should also be provided to the patients themselves, in lay format. Indeed the Transitions of Care Consensus Policy [16] advocates that patients, families and caregivers should have secure and private access to their information ("nothing about me, without me"). Patients should receive, understand and be encouraged to participate in the development of the transition record. They also specify that this process should take into consideration patients' health literacy and be culturally-sensitive. Providing patients with a summary of their baseline health status would empower patients to set their own goals both inside and outside the program, and may ultimately improve self-management. Empowering the patient with the tools to self-manage promotes active patient engagement in their health care, and along with support from PCPs, has been found to improve management of chronic health conditions and reduce health care costs [27].

### Limitations

Caution is warranted when interpreting results, chiefly due to response bias. First, female PCPs and those graduated from medical school more recently were significantly more likely to participate in the study than older, male PCPs. Second, given the low PCP response rate, the representativeness of the sample cannot be firmly established. However, we compared the sex of PCPs from our sample to the Canadian Medical Association master files (available online at http://www.cma.ca/index.cfm/ci_id/16959/la_id/1.htm#1) and found no significant differences in the percent of females between the study sample and national data (44.4% vs 38.5%; p = 0.17) [data not shown]. Overall, the relative homogeneity of physician samples has been established [28], lending credence to the generalizability of the findings. However, ultimately we cannot rule out the possibility that respondents had more favourable views towards CR.

Second, CR programs and PCPs were located in Ontario where some CR services are partially reimbursed through government, which may limit generalizability to other reimbursement systems (or lack thereof). However, the CR sites in this study were chosen to reflect a diversity of patient volumes, number of CR staff, length of program, degree of rurality, and whether the program was hospital or community-based to mitigate against this threat to generalizability. Finally, while each of the CR programs participating in this study provide services in accordance with Canadian guidelines [8], we cannot rule out diversity among programs which may influence providers' perspectives.

## Conclusion

In conclusion, this was, to the best of our knowledge the first study to systematically examine the important issue of CR program intake transition record generation, receipt and utility to PCPs of patients participating in CR. Less than half of PCPs receive CR intake transition records, although most desire them. PCPs are often informed by their patients that they have enrolled in CR, which renders them unable to reinforce and be consistent in patient recommendations and care plans implemented by the CR program. PCPs and patients alike should be provided with timely and evidence-based transition records, to support chronic disease management.

## Competing interests

The authors declare that they have no competing interests.

## Authors' contributions

JY and SG conducted the statistical analyses and wrote the initial drafts for this manuscript. KU, NS, RDR, and VJ participated in the design of the study and made essential revisions to the manuscript. All authors read and approved the final version of the manuscript.

## Pre-publication history

The pre-publication history for this paper can be accessed here:

http://www.biomedcentral.com/1472-6963/11/231/prepub
